# Supplementary material for: Machine learning identifies phenotypic profile alterations of human dopaminergic neurons exposed to bisphenols and perfluoroalkyls
Source: Sci Rep. 2023 Dec 11;13:21907. doi: 10.1038/s41598-023-49364-y (PMC10713827; doi:10.1038/s41598-023-49364-y)
Supplement: Supplementary file 1 — Supplementary Table 1. [file 41598_2023_49364_MOESM1_ESM.pdf]

Table S1. List of reagents, links to PhenoLink software and Python-based Jupyter notebook to perform data standardization, supervised classification, and plotting.

Name of Material/ Equipment	Company	Catalog Number
Anti- chicken - Alexa 647	Jackson ImmunoRearch	703-605-155
Anti-Map2	Novus	NB300-213
Anti-mouse - Alexa 488	Thermo Fisher	A11001
Anti-rabbit - Alexa 555	Thermo Fisher	A21429
Anti-Tyrosine Hydroxylase	Merck	T2928
Anti-α-synuclein	Abcam	138501
Bisphenol A (BPA)	Wellington Laboratories	BPA (80-05-7)
Bisphenol S (BPS)	Wellington Laboratories	BPS (80-09-1)
Bravo Automated Liquid Handling Platform with 384ST head	Agilent	
Confocal microscope	Yokogawa	CV7000
Countess Automated cell counter	Invitrogen	
DPBS +/+	Gibco	14040-133
EL406 Washer Dispenser	BioTek (Agilent)	
Formaldehyde Solution (PFA 16 %)	Euromedex	EM-15710-S
Hoechst 33342	Invitrogen	H3570
iCell Base Medium 1	Fujifilm	M1010
iCell DPN, Donor#01279, Phenotype AHN, lot#106339, 1M	Fujifilm	C1087
iCell Nervous System Supplement	Fujifilm	M1031
iCell Neural Supplement B	Fujifilm	M1029
Jupyter Python notebook for data analysis and plotting	In-house development	https://github.com/Ksilink/Notebooks/tree/main/Neuro/EndocrineDisruptorProfiling
Laminin	Biolamina	LN521
Perfluorooctane sulfonate (PFOS)	Wellington Laboratories	L-PFOS (4021-47-0)
Perfluorooctanoic acid (PFOA)	Wellington Laboratories	PFOA (335-67-1)
PFE-360	MedChemExpress	HY-120085
PhenoLink image segmentation software	In-house development	https://github.com/Ksilink/PhenoLink
PhenoPlate 384w, PDL coated	Perkin Elmer	6057500
Storage plates Abgene 120µL	Thermo Scientific	AB-0781
Triton	Sigma	T9284
Trypan Blue	Sigma	T8154-20ML
Vprep Pipetting System	Agilent	
