# Supplementary material for: Machine learning identifies phenotypic profile alterations of human dopaminergic neurons exposed to bisphenols and perfluoroalkyls
Source: Sci Rep. 2023 Dec 11;13:21907. doi: 10.1038/s41598-023-49364-y (PMC10713827; doi:10.1038/s41598-023-49364-y)
Supplement: Supplementary file 2 — Supplementary Table 2. [file 41598_2023_49364_MOESM2_ESM.pdf]

Table S2. Compositions of solutions and buffers.

Complete Maintenance Media	
**Reagents**	**Final conc.**
iCell Base Medium 1	0.97
iCell Neural Supplement B	0.02
iCell Nervous System Suppl.	0.01
**Blocking solution**	
**Reagent**	**Final conc.**
PBS 1X	1
Triton 10 %	0.001
FBS stock solution	0.1
**Primary staining solution**	
**Reagent**	**Final conc.**
PBS 1X	1
Triton 10 %	0.001
FBS stock solution	0.05
Anti-TH	1/1000
Anti-α-synuclein	1/500
Anti-MAP2	1/5000
**Secondary staining solution**	
**Reagent**	**Final conc.**
PBS 1X	1
Triton 10 %	0.001
FBS stock solution	0.05
Anti-mouse A488	1/1000
Anti-rabbit A555	1/1000
Anti-chicken A647	1/250
Hoechst	1/2000
