# Supplementary material for: Machine learning identifies phenotypic profile alterations of human dopaminergic neurons exposed to bisphenols and perfluoroalkyls
Source: Sci Rep. 2023 Dec 11;13:21907. doi: 10.1038/s41598-023-49364-y (PMC10713827; doi:10.1038/s41598-023-49364-y)
Supplement: Supplementary file 3 — Supplementary Table 3. [file 41598_2023_49364_MOESM3_ESM.pdf]

Table S3. The following table includes the 126 phenotypic features obtained.

	Feature name	Feature description
1	Cell_Correlation_MAP2_SNCA	Pearson correlation between MAP2 and SNCA channel
2	Cell_Correlation_TH_SNCA	Pearson correlation between TH and SNCA channel
3	Cell_Correlation_VanSteelselsMeanX_TH_SNCA	Van Steelsel's cross correlation between TH and SNCA channel, shift on x-axis
4	Cell_Correlation_VanSteelselsMeanY_TH_SNCA	Van Steelsel's cross correlation between TH and SNCA channel, shift on y-axis
5	Cell_Correlation_VanSteelselsSigmaX_TH_SNCA	SD of Van Steelsel's cross correlation between TH and SNCA channel, shift on x-axis
6	Cell_Correlation_VanSteelselsSigmaY_TH_SNCA	SD of Van Steelsel's cross correlation between TH and SNCA channel, shift on y-axis
7	Cell_Intensity_MeanIntensity_MAP2	Mean pixel intensity of MAP2 channel
8	Cell_Intensity_MeanIntensity_SNCA	Mean pixel intensity of SNCA channel
9	Cell_Intensity_MeanIntensity_TH	Mean pixel intensity of TH channel
10	Cell_Intensity_SumIntensityPerNuclei_SNCA	Integrated pixel intensity of SNCA channel normalized to number of nuclei
11	Cell_MAP2_SNCA_Intensity_MeanIntensity_SNCA	Mean pixel intensity of SNCA channel colocalized to MAP2 channel
12	Cell_Neurites_BranchingPointsPerNuclei_MAP2	Dendritic branching points of MAP2 channel normalized to number of nuclei
13	Cell_Neurites_BranchingPointsPerNuclei_TH	Dendritic branching points of TH channel normalized to number of nuclei
14	Cell_Neurites_LengthPerNuclei_MAP2	Dentritic network length of MAP2 channel normalized to number of nuclei
15	Cell_Neurites_LengthPerNuclei_TH	Dentritic network length of TH channel normalized to number of nuclei
16	Cell_Neurites_Length_MAP2	Dentritic network length of MAP2 channel
17	Cell_Neurites_Length_TH	Dentritic network length of TH channel
18	Cell_SurfacePerNuclei_MAP2	Surface pixels occupied by MAP2 channel, normalized to number of nuclei
19	Cell_SurfacePerNuclei_MAP2_SNCA	Surface pixels occupied by colocalized MAP2 and SNCA channel normalized to number of nuclei
20	Cell_SurfacePerNuclei_SNCA	Surface pixels occupied by SNCA channel, normalized to number of nuclei
21	Cell_SurfacePerNuclei_TH	Surface pixels occupied by TH channel, normalized to number of nuclei
22	Cell_SurfacePerNuclei_TH_SNCA	Surface pixels occupied by colocalized TH and SNCA channel normalized to number of nuclei
23	Cell_Surface_RatioSurface_TH_SNCA	Surface ratio occupied by colocalized TH and SNCA channel
24	Cell_Surface_TotalSurface_MAP2	Surface pixels occupied by MAP2 channel
25	Cell_Surface_TotalSurface_SNCA	Surface pixels occupied by SNCA channel
26	Cell_Surface_TotalSurface_SNCA_MAP2	Surface pixels occupied by colocalized SNCA and MAP2 channel
27	Cell_Surface_TotalSurface_TH	Surface pixels occupied by TH channel
28	Cell_Surface_TotalSurface_TH_SNCA	Surface pixels occupied by colocalized TH and SNCA channel
29	Cell_TH_SNCA_Intensity_MeanIntensity_SNCA	Mean pixel intensity of SNCA channel colocalized to TH channel
30	Cell_TH_SNCA_Intensity_MeanIntensity_TH	Mean pixel intensity of TH channel colocalized to SNCA channel
31	Cell_TH_SNCA_Intensity_SumIntensityPerNuclei_SNCA	Integrated pixel intensity of SNCA channel colocalized to TH channel normalized to number of nuclei
32	Cell_Texture_SNCA_AngularSecondMoment_000	Haralick uniformity of distribution of gray levels at 0 degree shift
33	Cell_Texture_SNCA_AngularSecondMoment_045	Haralick uniformity of distribution of gray levels at 45 degree shift
34	Cell_Texture_SNCA_AngularSecondMoment_090	Haralick uniformity of distribution of gray levels at 90 degree shift
35	Cell_Texture_SNCA_AngularSecondMoment_135	Haralick uniformity of distribution of gray levels at 135 degree shift
36	Cell_Texture_SNCA_Contrast_000	Haralick contrast of gray levels at 0 degree shift
37	Cell_Texture_SNCA_Contrast_045	Haralick contrast of gray levels at 45 degree shift
38	Cell_Texture_SNCA_Contrast_090	Haralick contrast of gray levels at 90 degree shift
39	Cell_Texture_SNCA_Contrast_135	Haralick contrast of gray levels at 135 degree shift
40	Cell_Texture_SNCA_Correlation_000	Haralick correlation of gray levels at 0 degree shift
41	Cell_Texture_SNCA_Correlation_045	Haralick correlation of gray levels at 45 degree shift
42	Cell_Texture_SNCA_Correlation_090	Haralick correlation of gray levels at 90 degree shift
43	Cell_Texture_SNCA_Correlation_135	Haralick correlation of gray levels at 135 degree shift
44	Cell_Texture_SNCA_DifferenceEntropy_000	Haralick difference of randomness of gray levels at 0 degree shift
45	Cell_Texture_SNCA_DifferenceEntropy_045	Haralick difference of randomness of gray levels at 45 degree shift
46	Cell_Texture_SNCA_DifferenceEntropy_090	Haralick difference of randomness of gray levels at 90 degree shift
47	Cell_Texture_SNCA_DifferenceEntropy_135	Haralick difference of randomness of gray levels at 135 degree shift
48	Cell_Texture_SNCA_DifferenceVariance_000	Haralick difference of variance of gray levels at 0 degree shift
49	Cell_Texture_SNCA_DifferenceVariance_045	Haralick difference of variance randomness of gray levels at 45 degree shift
50	Cell_Texture_SNCA_DifferenceVariance_090	Haralick difference of variance randomness of gray levels at 90 degree shift
51	Cell_Texture_SNCA_DifferenceVariance_135	Haralick difference of variance randomness of gray levels at 135 degree shift
52	Cell_Texture_SNCA_Entropy_000	Haralick randomness of gray levels at 0 degree shift
53	Cell_Texture_SNCA_Entropy_045	Haralick randomness of gray levels at 45 degree shift
54	Cell_Texture_SNCA_Entropy_090	Haralick randomness of gray levels at 90 degree shift
55	Cell_Texture_SNCA_Entropy_135	Haralick randomness of gray levels at 135 degree shift
56	Cell_Texture_SNCA_InfoMeasuresOfCorr1_000	Haralick information measure of correlation 1 of gray levels at 0 degree shift
57	Cell_Texture_SNCA_InfoMeasuresOfCorr1_045	Haralick information measure of correlation 1 of gray levels at 45 degree shift
58	Cell_Texture_SNCA_InfoMeasuresOfCorr1_090	Haralick information measure of correlation 1 of gray levels at 90 degree shift
59	Cell_Texture_SNCA_InfoMeasuresOfCorr1_135	Haralick information measure of correlation 1 of gray levels at 135 degree shift
60	Cell_Texture_SNCA_InfoMeasuresOfCorr2_000	Haralick information measure of correlation 2 of gray levels at 0 degree shift
61	Cell_Texture_SNCA_InfoMeasuresOfCorr2_045	Haralick information measure of correlation 2 of gray levels at 45 degree shift
62	Cell_Texture_SNCA_InfoMeasuresOfCorr2_090	Haralick information measure of correlation 2 of gray levels at 90 degree shift
63	Cell_Texture_SNCA_InfoMeasuresOfCorr2_135	Haralick information measure of correlation 2 of gray levels at 135 degree shift
64	Cell_Texture_SNCA_InverseDiffMoment_000	Haralick homogeneity of gray levels at 0 degree shift
65	Cell_Texture_SNCA_InverseDiffMoment_045	Haralick homogeneity of gray levels at 45 degree shift
66	Cell_Texture_SNCA_InverseDiffMoment_090	Haralick homogeneity of gray levels at 90 degree shift
67	Cell_Texture_SNCA_InverseDiffMoment_135	Haralick homogeneity of gray levels at 135 degree shift
68	Cell_Texture_SNCA_SumAverage_000	Haralick sum of averages of gray levels at 0 degree shift
69	Cell_Texture_SNCA_SumAverage_045	Haralick sum of averages of gray levels at 45 degree shift
70	Cell_Texture_SNCA_SumAverage_090	Haralick sum of averages of gray levels at 90 degree shift
71	Cell_Texture_SNCA_SumAverage_135	Haralick sum of averages of gray levels at 135 degree shift
72	Cell_Texture_SNCA_SumEntropy_000	Haralick sum of gray level randomness at 0 degree shift
73	Cell_Texture_SNCA_SumEntropy_045	Haralick sum of gray level randomness at 45 degree shift
74	Cell_Texture_SNCA_SumEntropy_090	Haralick sum of gray level randomness at 90 degree shift
75	Cell_Texture_SNCA_SumEntropy_135	Haralick sum of gray level randomness at 135 degree shift
76	Cell_Texture_SNCA_SumOfSquares_000	Haralick sum of square gray level variance at 0 degree shift
77	Cell_Texture_SNCA_SumOfSquares_045	Haralick sum of square gray level variance at 45 degree shift
78	Cell_Texture_SNCA_SumOfSquares_090	Haralick sum of square gray level variance at 90 degree shift
79	Cell_Texture_SNCA_SumOfSquares_135	Haralick sum of square gray level variance at 135 degree shift
80	Cell_Texture_SNCA_SumVariance_000	Haralick sum of gray level variance at 0 degree shift
81	Cell_Texture_SNCA_SumVariance_045	Haralick sum of gray level variance at 45 degree shift
82	Cell_Texture_SNCA_SumVariance_090	Haralick sum of gray level variance at 90 degree shift
83	Cell_Texture_SNCA_SumVariance_135	Haralick sum of gray level variance at 135 degree shift
84	Cytoplasm_Intensity_MeanIntensity_SNCA	Mean pixel intensity of cytoplasmic SNCA channel
85	Cytoplasm_MAP2_SNCA_Intensity_MeanIntensity_SNCA	Mean pixel intensity of cytoplasmic SNCA channel colocalized to MAP2 channel
86	Cytoplasm_SurfacePerNuclei_SNCA	Surface pixels occupied by cytoplasmic SNCA channel normalized to number of nuclei
87	Cytoplasm_SurfacePerNuclei_TH_SNCA	Surface pixels occupied by colocalized cytoplasmic TH and SNCA channel normalized to number of nuclei
88	Cytoplasm_Surface_TotalSurface_SNCA	Surface pixels occupied by cytoplasmic SNCA channel
89	IndividualCell_Intensity_MeanIntensity_SNCA	Mean pixel intensity of SNCA channel based on all individually segmented cells
90	IndividualCell_Intensity_RadialProfile_InterceptFit_SNCA	Fitted intercept of SNCA channel decay from center to edge
91	IndividualCell_Intensity_RadialProfile_MaxSlope_SNCA	Maximum steepness of SNCA channel decay from center to edge
92	IndividualCell_Intensity_RadialProfile_Maximum_SNCA	Maximum intensity of SNCA channel from center to edge
93	IndividualCell_Intensity_RadialProfile_MeanCoeffVar_SNCA	Mean SNCA channel dispersion from center to edge
94	IndividualCell_Intensity_RadialProfile_MeanGradient_SNCA	Mean shape of SNCA channel decay from center to edge
95	IndividualCell_Intensity_RadialProfile_Mean_SNCA	Mean intensity of SNCA channel from center to edge
96	IndividualCell_Intensity_RadialProfile_Median_SNCA	Median intensity of SNCA channel from center to edge
97	IndividualCell_Intensity_RadialProfile_Minimum_SNCA	Minimum intensity of SNCA channel from center to edge
98	IndividualCell_Intensity_RadialProfile_Q1_SNCA	First quartile intensity of SNCA channel from center to edge
99	IndividualCell_Intensity_RadialProfile_Q3_SNCA	Third quartile intensity of SNCA channel from center to edge
100	IndividualCell_Intensity_RadialProfile_SlopeFit_SNCA	Fitted slope of SNCA channel decay from center to edge
101	IndividualCell_Intensity_RadialProfile_StdCoeffVar_SNCA	SD of SNCA channel dispersion from center to edge
102	IndividualCell_Intensity_RadialProfile_Std_SNCA	SD of SNCA channel intensity from center to edge
103	IndividualCell_Intensity_SumIntensity_SNCA	Integrated pixel intensity of SNCA channel based on all individually segmented cells
104	IndividualCell_Surface_MeanSurface_SNCA	Mean surface pixels occupied by SNCA channel based on all individually segmented cells
105	Membrane_Intensity_MeanIntensity_MAP2	Mean pixel intensity of MAP2 channel on cellular edge
106	Membrane_Intensity_MeanIntensity_SNCA	Mean pixel intensity of TH channel on cellular edge
107	Membrane_Intensity_MeanIntensity_TH	Mean pixel intensity of MAP2 channel on cellular edge
108	Membrane_Surface_SurfacePerNuclei_MAP2	Surface pixels on cellular edge occupied by MAP2 channel normalized to number of nuclei
109	Membrane_Surface_SurfacePerNuclei_SNCA	Surface pixels on cellular edge occupied by SNCA channel normalized to number of nuclei
110	Membrane_Surface_SurfacePerNuclei_TH	Surface pixels on cellular edge occupied by TH channel normalized to number of nuclei
111	Nuclei_Living_Ratio_MAP2	Ratio of MAP2 channel positive nuclei
112	Nuclei_Living_Ratio_MAP2_SNCA	Ratio of MAP2 and SNCA channel positive nuclei
113	Nuclei_Living_Ratio_SNCA	Ratio of SNCA channel positive nuclei
114	Nuclei_Living_Ratio_TH	Ratio of TH channel positive nuclei
115	Nuclei_Living_Ratio_TH_SNCA	Ratio of TH and SNCA channel positive nuclei
116	Nuclei_Number_Big	Number of large nuclei
117	Nuclei_Number_Dead	Number of condensed/bright nuclei
118	Nuclei_Number_Living	Number of nuclei based on Hoechst channel
119	Nuclei_Number_MAP2	Number of MAP2 channel positive nuclei
120	Nuclei_Number_MAP2_SNCA	Number of MAP2 and SNCA channel positive nuclei
121	Nuclei_Number_SNCA	Number of SNCA channel positive nuclei
122	Nuclei_Number_TH	Number of TH channel positive nuclei
123	Nuclei_Number_TH_SNCA	Number of TH and SNCA channel positive nuclei
124	Nuclei_Ratio_Dead	Ratio of condensed/bright nuclei
125	Nuclei_Ratio_Living	Ratio of nuclei not considered condensed/bright
126	Nuclei_Surface_MeanArea	Mean surface pixels of Hoechst channel

MAP2 = microtubule-associated protein 2; TH = tyrosine hydroxylase; SNCA = alpha-synuclein.
